# *In vivo* tissue optical clearing assisted through-skull targeted photothrombotic ischemic stroke model in mice

**DOI:** 10.1117/1.JBO.27.6.065001

**Published:** 2022-06-08

**Authors:** Zhengwu Hu, Dongyu Li, Xiang Zhong, Yusha Li, Ang Xuan, Tingting Yu, Jingtan Zhu, Dan Zhu

**Affiliations:** a Huazhong University of Science and Technology, Britton Chance Center for Biomedical Photonics, Wuhan National Laboratory for Optoelectronics, Hubei, Wuhan, China; b Huazhong University of Science and Technology, MoE Key Laboratory for Biomedical Photonics, Hubei, Wuhan, China; c Optics Valley Laboratory, Hubei, China

**Keywords:** ischemic stroke, skull optical clearing, laser speckle contrast imaging, photothrombosis

## Abstract

**Significance:**

Photothrombotic stroke is an important and widely used model for ischemic stroke research. However, the significant scattering of the skull during the procedure limits the light’s ability to penetrate and focus on its target. Targeted photothrombosis uses surgery-based skull windows to obtain optical access to the brain, but it renders the brain’s environment unnatural even before a stroke is established.

**Aim:**

To establish a targeted, controllable ischemic stroke model in mice through an intact skull.

**Approach:**

The *in vivo* skull optical clearing technique provides a craniotomy-free “optical window” that allows light to penetrate. Alongside the local photodynamic effect, we have established targeted photothrombosis without skull removal, effectively controlling the degree of thrombotic occlusion by changing the light dose.

**Results:**

*Ex vivo* and *in vivo* results demonstrated that skull optical clearing treatment significantly enhanced light’s ability to penetrate the skull and focus on its target, contributing to thrombotic occlusion. The skull optical clearing window was also used for continuous blood flow mapping, and the relationship between light dose and injury degree was evaluated over 14 days of monitoring. Per our findings, increasing the light dose was accompanied by more severe infarction, indicating that the model was easily controllable.

**Conclusions:**

Herein, a targeted, controllable ischemic stroke model was established by combinedly running an *in vivo* skull optical clearing technique and a photothrombotic procedure, avoiding unnecessary damage or environmental changes to the brain caused by surgery on the skull. Our established model should offer significant value to research on ischemic stroke.

## 1 Introduction

Stroke is a cerebrovascular disease with high rates of fatality, disability, and recurrence.^1^^–^^3^ It occurs as hemorrhagic or ischemic, with ischemic stroke accounting for 70% of cases and causing brain tissue damage and neurological impairment that result in a range of sequelae.^4^ Consequently, ischemic stroke has attracted mounting attention in modern biomedical research.^4^^–^^7^

Multiple animal models,^4^^,^^8^ including the intraluminal suture model,^9^^,^^10^ FeCl3-induced model,^11^ photothrombotic model,^12^ and *in situ* thrombin injection model,^13^ have been established to explore the pathogenesis of ischemic stroke, screen effective therapeutic drugs, and seek rational treatment options. The photothrombotic model uses a local photodynamic effect and is preferred for its easy control of the location and extent of thrombotic occlusion.

The photothrombotic process consists first of intraperitoneally or intravenously injecting photosensitizers into an animal’s circulatory system, where they bind to vascular endothelial cells, platelets, and other blood cells. These photosensitizers then, under local laser irradiation with a specific wavelength, produce reactive oxygen species and inhibit the activity of oxygen radical scavengers, leading to the peroxidation of the vascular lining, adhesion, recruitment of platelets, and ultimately the formation of thrombi, alongside a variety of intravascular adhesion factors at the irradiated region.^14^^,^^15^ Hence, a photothrombotic stroke positioning is determined by the site of laser irradiation, and the severity is dictated by the dosage of a photosensitizer and exposure to light. However, the turbid skull above the cerebral cortex has an extremely high degree of scattering, which not only leads to a significant attenuation of the light but also renders light reaching the cortex difficult to collimate or focus, limiting the success rate and localization of the photothrombotic model.

To overcome the effect of the skull and establish targeted photothrombotic models, open-skull window and thinned-skull window techniques have been regularly used to attain vascular distribution in the cortex and thus select the target vessels or regions.^16^^–^^19^ However, surgical procedures on the skull often change the intracranial pressure and are prone to cause bleeding or inflammation during operations,^20^ creating an unnatural brain environment before a stroke is even established. Fortunately, the recently developed transcranial window and *in vivo* skull optical clearing technique could help avoid these problems by enabling the creation of photothrombotic models based on an intact skull.

The transcranial window provides a chronic “clear skull cap” by covering it with a layer of dental cement or clear nail polish. This chronic optical window method enables the imaging of an entire cerebral cortex for up to 2 months.^21^^–^^27^ Unlike the transcranial window with an optically transparent reagent covering the skull, the *in vivo* skull optical clearing dissociates the collagen from the skull and removes the lipid and calcium ions using urea ethanol solution, glycerol, sodium dodecyl benzene sulfonate (SDBS), and ethylene diamine tetraacetic acid applications, thus changing the composition of the skull and making it transparent without craniotomy. Washing the skull with phosphate buffered saline (PBS) enables it to regain its initial turbid status. In this way, the skull optical clearing technique, compared to transcranial windows, causes less scattering of the skull and, therefore, provides a synaptic imaging resolution. In addition, an *in vivo* skull optical clearing window is switchable and suitable for long-term brain observations for up to 7 months.^28^^,^^29^ Employing the skull optical clearing window, deep-tissue cortical vascular observation and superficial neurodevelopment monitoring (with dendritic spine resolution) have been realized before.^28^ The skull optical clearing window has also proven efficient in through-skull photodynamic blood–brain barrier opening and laser-induced targeted damage.^29^^–^^32^

In this investigation, combining the photothrombotic model with the skull optical clearing window ensured the development of a targeted ischemic stroke model through an intact skull. First, *ex vivo* experiments were performed to evaluate the enhanced ability of the skull optical clearing process to focus light through the skull. Second, laser speckle contrast imaging (LSCI) was used to assess the stability of the skull optical clearing window-induced enhancement of the through-skull photothrombotic model establishment. Third, after photothrombosis, blood flow distribution was continuously observed (6 h) using the optical clearing skull window to scrutinize the development of vascular endothelial injury-induced cerebral infarction. Additionally, the impact of different light dosages on the skull optical clearing window-assisted photothrombosis was evaluated through 14 days of tracking to provide a dose reference for the use of this model. This investigation offers an innovative approach for establishing a low-invasive targeted ischemic stroke model; it is hugely promising in fundamental research and drug development on ischemic stroke.

## 2 Materials and Methods

### 2.1 Animals

Male BALB/C mice and $\mathrm{CX}3\mathrm{CR}1^{\mathrm{EGFP}+/-}$ (8 weeks, Wuhan University Animal Experiment Center) were housed in a specific pathogen-free environment at the Wuhan Optoelectronics National Experimental Center, Huazhong University of Science and Technology. Animal husbandry, care, and experimental procedures were conducted strictly in accordance with the Hubei Provincial Experimental Animal Regulations.

### 2.2 Skull Optical Clearing Procedure

The switchable skull optical clearing window was established as reported earlier. Two solutions were used for the skull optical clearing procedure. Solution 1 (S1) was a saturated supernatant of ethanol (Sinopharm, China) and urea (Sinopharm, China) with a volume to mass ratio of 10:3 and an ethanol volume ratio of 75%. Solution 2 (S2) was a highly concentrated solution of SDBS. The key steps are as follows: (1) anesthetizing the mice with a cocktail of 2% α-chloralose and 10% urethane (8  mL/kg) via intraperitoneal injection; (2) cutting open mice scalps to expose the skull surface; (3) fixing holders to mice skulls with glue and then attaching them to custom plates; (4) adding S1 dropwise to the skull surface for about 10 min; (5) removing S1 and covering the skull with S2 for 5 min; (6) performing cortical imaging on the now transparent skull; (7) wiping off S2 after imaging and washing the skull with saline to return it to its original turbid state; and (8) removing the optical clearing agent and suturing the scalp, leaving the mice to recover from the anesthesia after 2 to 3 h. In the *ex vivo* experiments, the removed fresh skull was immersed in S1 for 10 min, wiped dry, and immersed in S2 for 5 min to prepare it for the optical clearing procedure. This description constitutes the entire procedure for skull optical clearing. The sham group was only anesthetized without scalp-opening operation and optical clearing treatment.

### 2.3 Photothrombosis Establishment through the Skull Optical Clearing Window

**Fig. 1:**
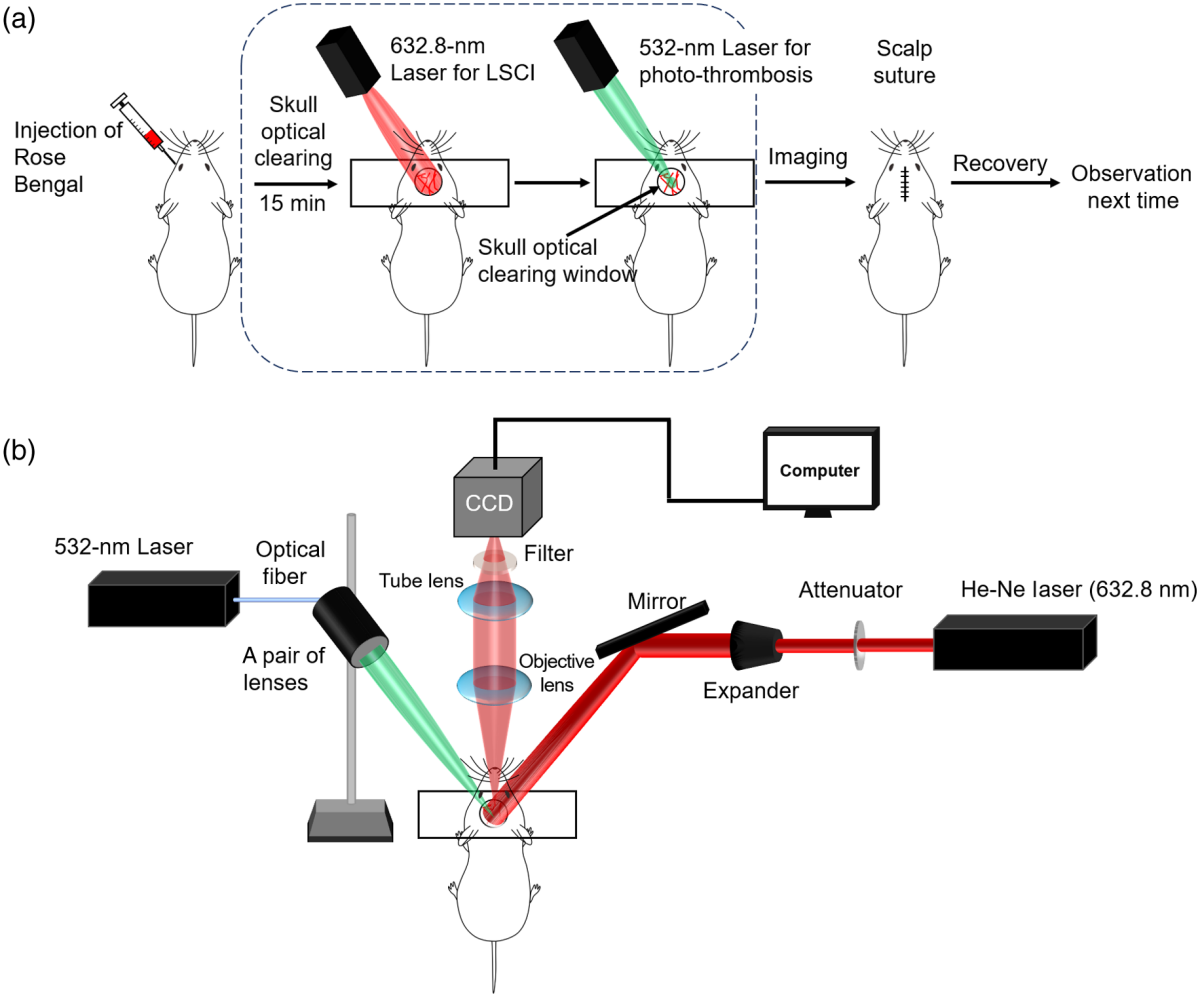
Schematic diagram of the experimental procedure (a) and optical system (b). A pair of lenses was used to focalize the 532-nm laser onto the cortex.

We evaluated the possibility of combining the photothrombosis technique with the *in vivo* skull optical clearing procedure to establish a through-skull, degree controllable, targeted ischemic stroke in mice. As shown in Fig. 1(a), mice were intravenously injected with 100  μL of Rose Bengal (25  mg/kg, Sigma-Aldrich, China) solution through the inner canthus after anesthesia before the establishment of the skull optical clearing window; therefore the subcarinal vessels were clearly visible. Cerebral blood flow distribution before stroke creation was determined using an LSCI system equipped with a He-Ne laser (632.8 nm, 3 mW), and the region of interest was irradiated with a 532-nm laser ($7\;\mathrm{mW}/{\mathrm{mm}}^{2}$, Changchun New Industry Optoelectronic Technology Co., Ltd., China) for 1, 2, or 5 min to induce cerebral infarction in the focused region at different degrees.

### 2.4 Laser Speckle Contrast Imaging of Cerebral Blood Flow

Change in cerebral blood flow velocity was monitored using a home-built LSCI system. As shown in Fig. 1(b), a He-Ne laser (632.8 nm, 3 mW) was passed through a collimated diffusion beam mirror and then uniformly irradiated to the target imaging area, and the resulting backscattered light was captured with a camera (Pixelfly, PCO GmBH©, Germany) after passing through a stereomicroscope (SZX7, Olympus, Japan) and recorded using a computer (40 frames, exposure time 20 ms). The original scattered images were processed through temporal contrast analysis to obtain blood flow distribution, which is expressed as^33^^,^^34^

$$K_{t_{\left(x,y\right)}}=\frac{\sigma_{\left(x,y\right)}}{\left\langle I_{\left(x,y\right)}\right\rangle },$$

where $K_{t_{\left(x,y\right)}}$ is the temporal contrast at pixel (x,y), $\sigma_{\left(x,y\right)}$ is the standard deviation of pixel intensity corresponding to this coordinate in 40 images, and $\left\langle I_{\left(x,y\right)}\right\rangle$ is the average pixel intensity corresponding to this coordinate in 40 images. $1/K_{t}^{2}$ reflects the speed of blood flow. Although the calculated cerebral blood flow (CBF) was a relative value instead of an absolute one, it reflected changes in CBF under the same imaging condition.

1× and 2.5× magnifications were used to capture the bi-hemisphere and local region, respectively: the fields of view were 9  mm×6.7  mm and 3.6  mm×2.7  mm, respectively.

### 2.5 Immunofluorescence

The brain vasculature and neuronal nuclei were stained with Dylight 649 *L.esculentum* (tomato) lectin (LEL-Dylight649, DL-1178, Vector Laboratories) and an anti-NeuN antibody (Abcam, United Kingdom, ab177487), respectively. Mice were perfused with paraformaldehyde (PFA) 2 min after the intravenous injection of LEL-Dylight 649 (0.1  mg/mL, 100  μL), and their brains were removed, fixed in 4% PFA at 4°C overnight, embedded in 4% agarose (Sigma-Aldrich, St. Louis, Missouri, United States), and sliced coronally into 100  μm-thick sections using a vibratome (Leica VT1000, Germany). These sections were subsequently washed three times (5 min each time) with PBS-Triton (PBST, 0.2% Triton X-100 in PBS), incubated in a blocking solution (5% normal goat serum/PBST) for 1 h, incubated with an anti-NeuN antibody in 5% normal goat serum/PBST overnight at 4°C and then at room temperature for 1 h, washed five times (5 min each time), incubated with 0.2% goat anti-rabbit-AF555 antibody (Invitrogen, United States, A-21428) for 5 h, washed five times (5 min each time), and imaged with a commercial confocal microscope (LSM710; Zeiss, Oberkochen, Germany).

### 2.6 Infarct Volume Measurement

Anti-NeuN-stained brain sections were imaged with a confocal microscope, and the maximum projection was obtained for each brain section. Areas that remained unstained were considered infarcts. The infarct area $S_{\text{infarct}}$ of each anti-NeuN-stained coronal surface brain slice with a thickness of h (100  μm) was measured with ImageJ (Bethesda, Maryland, United States). The infarct volume was obtained by determining the sum of the volumes of each infarcted brain slice, that is $V_{\text{infarct}}=\mathrm{SUM}(S_{\text{infarct}}\times h)$.

### 2.7 Statistical Analysis

Data are expressed as the mean ± standard error and were analyzed using Statistical Product Service Solutions software (SPSS, IBM, United States). One-way analysis of variance (ANOVA) was used to compare more than two groups. Values were considered significant at P<0.05 (*P<0.05, **P<0.01, and ***P<0.001).

## 3 Results

### 3.1 Establishment of Cortical Photothrombosis through the Skull Optical Clearing Window

**Fig. 2:**
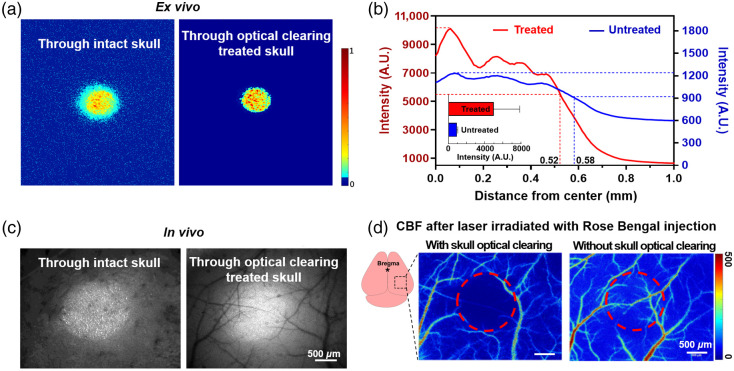
Enhancement of light transmission after skull optical clearing treatment. (a) Representative normalized images of 532-nm laser spots through skull samples before and after optical clearing treatment. The color represents the recorded intensity, which was normalized against the highest intensity. (b) Light intensity distribution from the center in (a). (c) Representative *in vivo* photographs of a skull-covered brain’s subjection to the 532-nm laser irradiation. It was captured using the stereomicroscope under a bright field, with the 532-nm laser focused on the cortex. (d) LSCI-assisted CBF images after Rose Bengal injection and 532-nm laser irradiation for 5 min. The irradiation location was the same (AP=−2  mm, ML=2  mm) for each mouse. For the skull optical clearing group, irradiation was introduced after skull optical clearing window establishment, followed by LSCI detection. For the control group, irradiation was performed first, followed by skull optical clearing window establishment and LSCI detection. The color represents relative blood flow velocity. The red circles represent the laser irradiation area. Mean ± SD; n=3 per group.

To assess the turbid skull-caused limitation of the photothrombotic model and the skull optical clearing-induced improvement, both *in vivo* and *ex vivo* experiments were performed. First, we passed focused light (the radius of the light spot was 0.5 mm) through a mouse skull sample and imaged the result with a charge-coupled device (CCD). As shown in Fig. 2(a), the energy of light passing through the optical clearing treated skull was highly concentrated than that through the original cloudy skull. Figure 2(b) depicts the examination of all the detected signals through the skull under different conditions. Quantitative analysis demonstrated that the distance from the central position to the site where intensity drops by half when the laser passed through the turbid skull was 0.58 mm; it was 0.52 mm after optical clearing treatment [Fig. 2(b)], suggesting that the optical clearing treatment widened the 0.5-mm radius spot 12% less as the light beam passed through the original skull. Figure 2(b) indicated that the total laser power through the optical clearing treated skull was five times stronger as it went through the original skull.

*In vivo* experiments were used to evaluate the enhancement of skull optical clearing in establishing the photothrombotic model. As shown in Fig. 2(c), before skull optical clearing, we only observed the 532-nm light spot on the skull and were not aware of the cerebrovascular distribution under it. In contrast, the skull optical clearing window allowed for easy selection of areas or vessels to be occluded without opening the skull. As Fig. 2(d) shows, blood flow in the exposed regions decreased remarkably after the 532-nm laser irradiation through the skull optical clearing window, but the same irradiation dosage yielded no observable infarction through the original turbid skull, pointing to enhancement in the skull optical clearing window-induced photothrombotic efficacy.

**Fig. 3:**
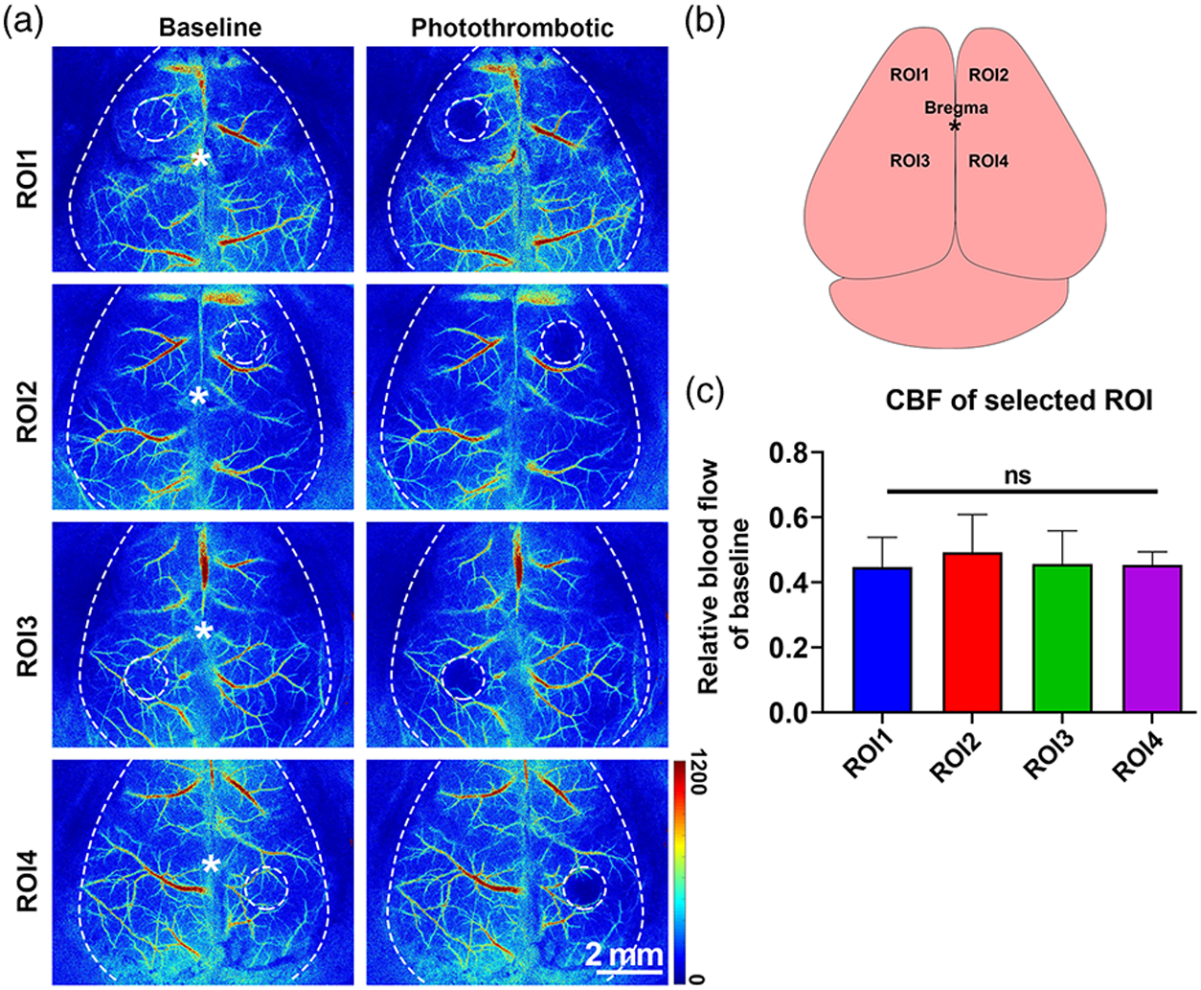
Evaluation of blood flow changes in selected areas of interest in the large field of view of the cerebral cortex for stroke. (a) Four regions based on the relative position of bregma in the cerebral cortex. The diameter of the ROI is 1 mm. (b) Diagram of the four regions. (c) Changes in blood flow in the four regions after stroke. Mean ± SD; ns—P>0.05, n=3 per group. ROI, region of interest.

We next selected four regions on the bi-hemispheres of the mice and established a targeted photothrombotic stroke, as shown in Fig. 3. The four regions correspond to the upper left, upper right, lower left, and lower right regions of the bregma [Fig. 3(b)]. The diameter of the light spot was 1 mm, and blood flow after stroke establishment with 5-min laser irradiation is demonstrated in Fig. 3(c). Blood flow in groups region of interest (ROI) 1, ROI 2, ROI 3, and ROI 4 decreased to 0.44±0.07, 0.49±0.09, 0.45±0.08, and 0.45±0.03, respectively, relative to baseline blood flow. Such results suggest that the turbid skull can limit the targeting ability and consistency of the photothrombotic model, whereas the skull optical clearing technique provides a craniotomy-free window for a stable model of ischemic strokes in regions of interest.

#### 3.2 Continuous Monitoring of CBF Post-Skull Optical Clearing Window-Induced Stroke

**Fig. 4:**
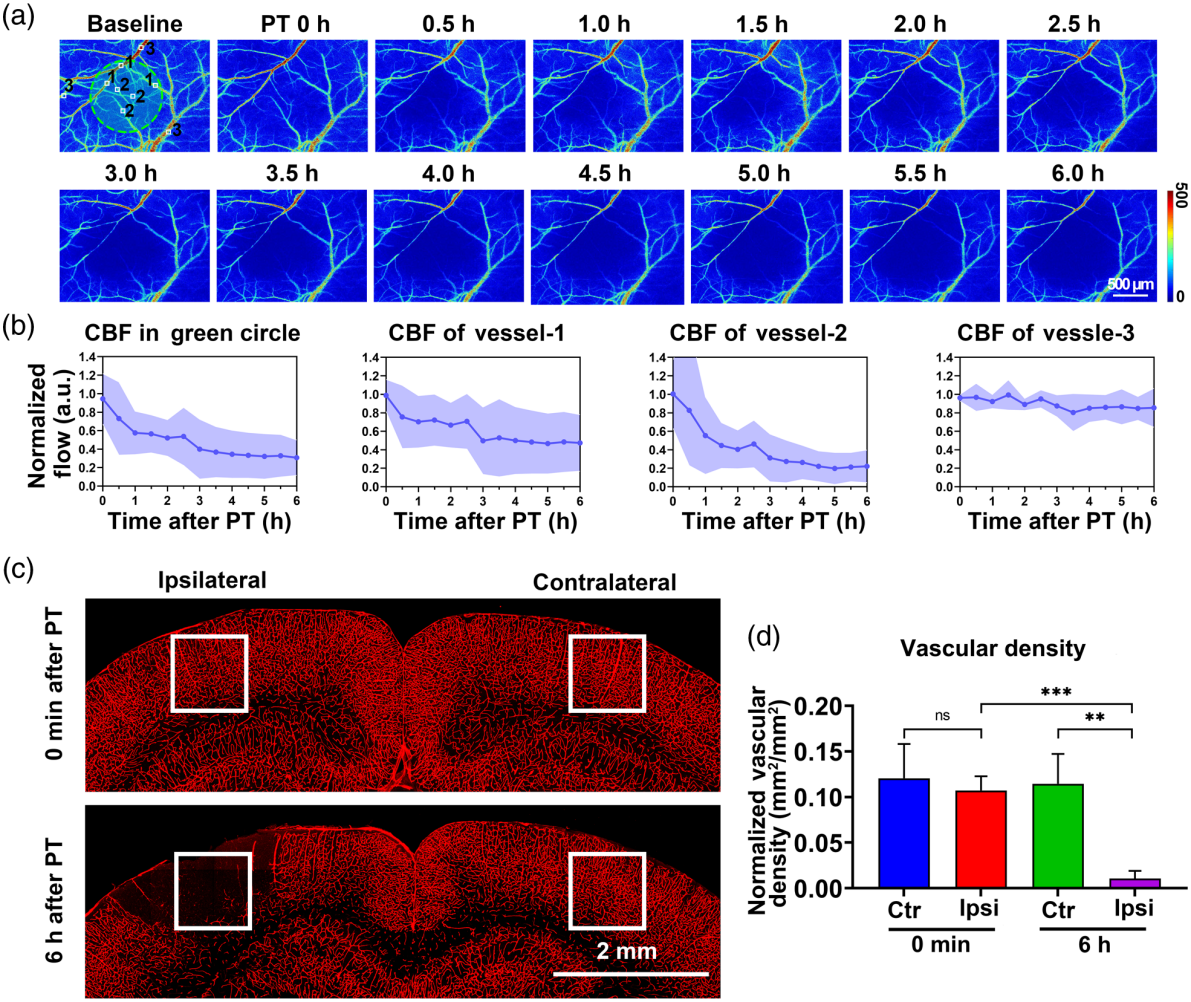
The 6-h observation after skull optical clearing window-assisted targeted photothrombosis. (a) Blood flow monitoring after the 2-min laser exposure. The diameter of the laser spot was 1 mm and is denoted by the green circle. Vessel-1 and vessel-2 represent relatively large and small vessels, respectively, within the laser-irradiated area, while vessel-3 is the vessel outside the area. (b) Changes in CBF in the green circle, vessel-1, vessel-2, and vessel-3 for 6 h after PT. CBF values were normalized against the baseline value (CBF value before PT). (c) Representative *ex vivo* coronal brain sections of mice (where the photothrombosis center was located) at 0 min and 6 h post-stroke, labeled with LEL-649 and imaged with a commercial confocal microscope (LSM710, Zeiss, Germany). (d) The density of blood vessels in the ischemic region. 1  mm×1  mm ROIs in the ischemic region and contralateral side were selected [white frames in (c)], where vasculature was binarized. Then, the ratio of vascular area to total ROI was calculated as the vascular density. Mean ± SD; ns—P>0.05, ***P<0.001; **P<0.01, n=3 per group. PT, photothrombosis.

Using the skull optical clearing technique, we not only established a targeted photothrombotic model but also tracked the changes in CBF distribution after stroke. Figure 4(a) shows CBF levels after Rose Bengal injection and laser irradiation for 6 h. Blood flow changes in the irradiated region and in the vessels inside and outside the region were measured as in Fig. 4(b). CBF in the laser-irradiated area gradually decreased during the 6 h of irradiation, dropping to 50% at 2 h post-stroke and eventually to about 30%. Similarly, blood flow in the relatively larger vessels in the region (indicated as number 1) dropped to about 50% within 3 h after the stroke but remained constant for the next 3 h. The drop in relatively smaller vessels in the region (indicated as number 2) was more rapid, reaching 50% after 1.5 h and about 20% after 6 h post-stroke. On the contrary, blood flow in the vessels outside the irradiated region (indicated as number 3) showed a slow and small decrease after 6 h. In addition to the *in vivo* observation of blood flow changes in the superficial layer, an *ex vivo* evaluation of the vascular blockage in the deep cortex was performed 6 h post-stroke [Figs. 4(c) and 4(d)]. The results display no significant difference in vessel density between the two sides of the cerebral cortex (0.12±0.03 versus 0.10±0.01) right after the 2-min laser irradiation. After 6 h, there was a significant infarction on the ipsilateral side, and the vessel density decreased to 0.01±0.00, while the density on the contralateral side remained 0.11±0.03. These results suggest that the extent of vascular damage after 2 min of irradiation is progressively amplified over 6 h.

### 3.3 Long-Term Monitoring of Skull Optical Clearing Window-Assisted Photothrombotic Model at Different Light Doses

**Fig. 5:**
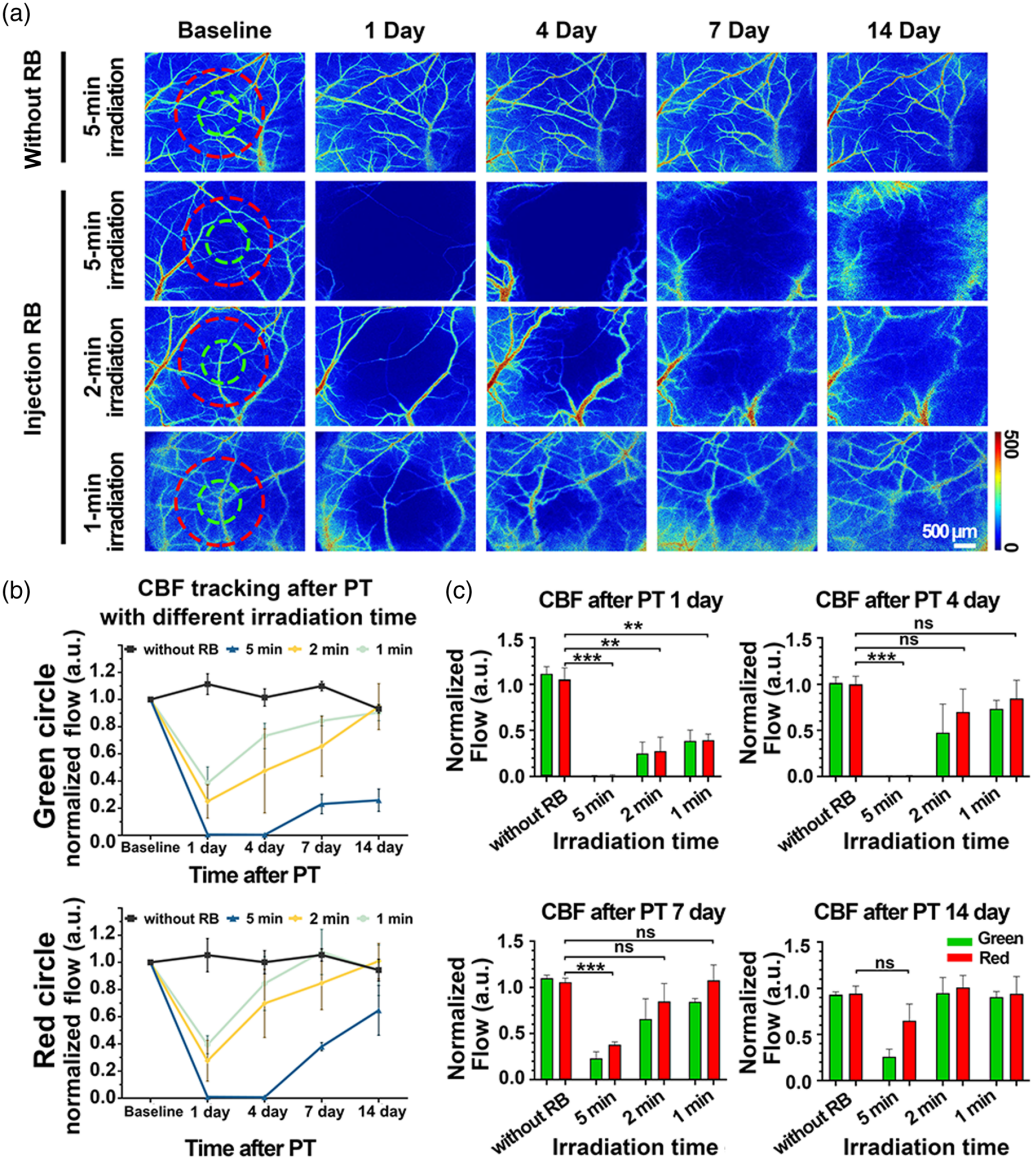
Cerebral blood flow over 14 days after photothrombosis establishment with various light doses. (a) Representative LSCI images of skull optical clearing window-engendered cortical blood flow for 14 days after photothrombosis. Green circles represent the positions of the 532-nm laser spots. (b) Changes in blood flow intensity with various light doses for 14 days in the green and red circles in (a). The values were normalized against the baseline. (c) CBF in the red circle in (a) on days 1, 4, 7, and 14 post-stroke at different irradiation doses. Mean ± SD; ns—P>0.05, ***P<0.001; **P<0.01, n=3 per group. RB, Rose Bengal; PT, photothrombosis.

In addition to scrutinizing blood flow changes in a short period, we tracked blood flow for a long period of up to 14 days to understand the occurrence of injury after stroke. And the light time was adjusted as a control to obtain different degrees of damage to the vascular injury. As shown in Fig. 5(a), 5 min of laser irradiation without Rose Bengal injection did not affect blood flow, indicating that such a light dose itself was safe for animals. On the contrary, alongside the Rose Bengal injection, the lengthier the exposure to light, the more significant the post-stroke damage induced. To make this clearer, we examined blood flow changes in the 1-mm light spot region [green circles in Fig. 5(a)] and a circle of 2 mm diameter centered on the light spot [reds circle in Fig. 5(a)]. Figures 5(b) and 5(c) show that blood flow fluctuated above and below the baseline when irradiated for 5 min in the absence of Rose Bengal injection. However, combinedly administering Rose Bengal and irradiation at different exposure times resulted in the fall and then rise of blood flow during the 14 days of scrutiny, with blood flow in the light spot region recovering slowly. To be specific, there were no vascular flow signals within the red circles in the 5-min-irradiation group, whose relative baseline blood flow average was 0.00±0.00, on day 1 post-stroke. On the other hand, we noted significant flow in the blood vessels in the 2-min-irradiation group. Besides, blood flow signals outside distinguishable vessels were also higher than zero, and the mean value was 0.27±0.12. In the 1-min group, the mean blood flow value was 0.39±0.05. On day 4 post-stroke, CBF in the 5-min irradiation group was still 0.00±0.00, but CBF in the 2- and 1-min irradiation groups increased to 0.69±0.20 and 0.84±0.16, respectively: they were not significantly different from the control group values (1.00±0.06, p=0.11 versus group 2-min, p=0.27 versus group 1-min). On day 7 post-stroke, CBF in the 5-min irradiation group increased to 0.38±0.02; CBF in the 2- and 1-min irradiation groups were 0.84±0.15 and 1.07±0.13, respectively. By day 14, CBF had recovered to 0.64±0.14 in the 5-min group, 1.00±0.10 in the 2-min group, and 0.94±0.15 in the 1-min group, with no significant difference between each injury group and the control group (0.94±0.06). Such results indicate that mice experienced different degrees of damage after exposure to different light doses in the skull optical clearing window procedure, and the two correlated positively. Blood flow further spontaneously recovered over the next 2 weeks, with the recovery ability correlating negatively with the light dose.

**Fig. 6:**
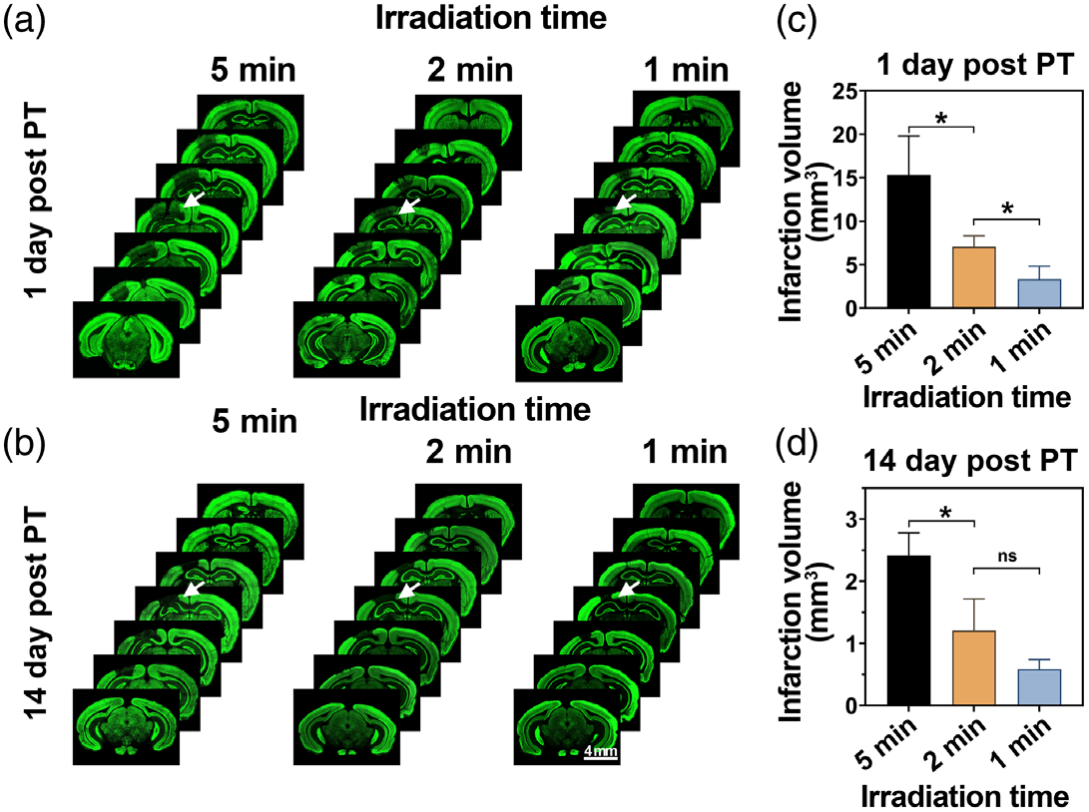
Infarct volumes on days 1 and 14 after photothrombosis establishment with various light doses. (a) Coronal sections of the brain on day 1 post-stroke under the 5-, 2-, and 1-min irradiation, all spaced 500  μm apart, where neurons were labeled with an anti-NeuN antibody. (b) Coronal sections of the brain on day 14 post-stroke under the 5-, 2-, and 1-min irradiation. The white arrows in (a) and (b) indicate stroke regions. (c) Infarct volumes 1 day after photothrombosis establishment. (d) Infarct volumes 14 days after photothrombosis establishment. Mean ± SD; ns—P>0.05, *P<0.05, n=3 per group.

To verify the aforementioned conclusions, we also counted the infarction volumes under different light doses (Fig. 6). The neurons were labeled with an anti-NeuN antibody and the vessels with LEL-649 (Fig. S1 in the Supplemental Material), and both were merged together. The results post-stroke day 1 with 5-, 2-, and 1-min laser irradiation are shown in Fig. 6(a), and those after 14 days of stroke are presented in Fig. 6(b). Blood flow in the LEL-649 channel recovered remarkably 14 days after stroke, which was consistent with *in vivo* findings (Fig. S1 in the Supplemental Material). Infarct volume statistics are based on neuronal staining. As shown in Figs. 6(c) and 6(d), the infarct volume in the 5-min irradiation group was the largest, reaching $15.29\pm 4.49\;{\mathrm{mm}}^{3}$ post-stroke day 1. The infarct volume in the 2-min irradiation group was $7.06\pm 1.23\;{\mathrm{mm}}^{3}$, only half of that of the 5-min irradiation group. The infarct volume in the 1-min irradiation group was the smallest, only $3.31\pm 1.50\;{\mathrm{mm}}^{3}$. After 14 days, the infarct volume in each group dropped considerably: $2.41\pm 0.36\;{\mathrm{mm}}^{3}$, $1.20\pm 0.50\;{\mathrm{mm}}^{3}$, and $0.58\pm 0.15\;{\mathrm{mm}}^{3}$ in the 5-, 2-, and 1-min irradiation groups, respectively.

Herein, the degree of injury in our stroke model is controllable: the longer the duration of exposure to light, the more severe the injury. CBF in mice post-stroke fell and then rose, and the infarct volume diminished, indicating that the mice recovered by themselves.

## 4 Discussion

We have shown that blood vessel distribution in the cortex can be seen clearly using the skull optical clearing window when establishing a targeted photothrombotic model without craniotomy, and infarction is controllable by simply adjusting the light dose. Stroke was induced in the targeted region with a simple irradiation system, which focused light into a 1-mm spot. If the focus of the laser is smaller, a more specific target would be blocked through our skull optical clearing window. Watts et al.^16^ used an objective lens to generate a tiny irradiation spot on the cerebral cortex through a thinned-skull window to achieve single vessel ischemic stroke. Clark et al.^35^ developed an artery-targeted photothrombotic model using a digital micromirror device with the skull removed. Sunil et al.^17^ established a targeted photothrombotic model in awake mice using optimized light patterns with a chronic open-skull glass window. Therefore, using such optimized optical systems, similar models could be undoubtedly established utilizing the skull optical clearing window.

In our results, we noted infarction quite clearly 1 day after either 1- or 2-min laser exposure post-Rose Bengal injection, [Fig. 5(a)]. However, an observable ischemic stroke occurred right after the 5-min irradiation [Fig. 2(d)], and thrombotic occlusion was not apparent after the 2-min exposure to light, gradually emerging after prolonged observation [Fig. 4(a)]. Such a difference is perhaps due to the different light doses. In the photothrombotic model, thrombi are formed because of the vascular endothelial cell injury caused by photosensitizer-produced reactive oxygen species.^36^^,^^37^ Therefore, for severe vascular endothelial cell injuries, platelets would aggregate rapidly to form thrombi, while for relatively lighter injuries, infarction would generate slowly.^14^ The thrombotic occlusion area could continue to expand for some time after occlusion, and the infarction region could exceed the irradiation spot area 1 day after occlusion because the blocked vessels would affect the connected vessels. Our results also showed that the blood vessels recanalized or regenerated after the peak of cerebral occlusion in mice, which was also observed in the open-skull window-established photothrombotic model.^38^^–^^40^

Past studies combined the transcranial window with photothrombosis in awake mice.^41^ Balbi et al. established focal ischemic stroke in cortex-targeted brain regions of awake head-fixed mice via a combination of *in vivo* bi-hemispheric transcranial windows and photothrombosis, observing the outcome longitudinally on a mesoscopic scale. The study demonstrated the effects of the cortical spreading of ischemic depolarization after focal ischemic stroke in anesthetized and awake mice, which has important implications for revealing changes in brain dynamics after stroke. In our study, we combined the *in vivo* skull optical clearing procedure with photothrombosis in the targeted area to establish a focal ischemic stroke and then evaluated blood flow changes after this stroke model at different infarct levels using laser speckle contrast imaging. In comparison, although the switchable *in vivo* skull optical clearing window is less convenient than chronic transcranial windows, it improves the resolution (spine-resolution)^28^ of cortical imaging and the depth (up to 900  μm)^42^ of cerebral vascular and neural imaging without craniotomy, facilitating our ability to observe subcortical cerebral vascular and neurosynaptic damage and repair post-stroke in future investigations.^28^^,^^29^

To evaluate the impact of repetitively exposing mice skulls to optical clearing, we used $\mathrm{CX}3\mathrm{CR}1^{\mathrm{EGFP}+/-}$ mice to observe microglia after 14 days of cortical imaging (optical clearing was performed on days 1, 4, 7, and 14 post-stroke) and found that the microglia were not activated, indicating that there was no immune response in the brain (Fig. S2 in the Supplemental Material). The bodyweights of the mice were also recorded, and the results also show no significant differences between the experimental and sham groups (Fig. S3 in the Supplemental Material).

The anesthesia scheme used in this research is commonly applied in animal experiments. However, it is more commonly employed in terminal procedures instead of longitudinal studies. Therefore, while it had no influence on the conclusion that a controllable targeted ischemic stroke model was established through an intact skull using an *in vivo* optical clearing technique, it is more appropriate to use other anesthetics, such as isoflurane, for longitudinal observations in future studies.

Our injection of Rose Bengal 20 min before laser irradiation was to ensure stabilization of the Rose Bengal concentration in the blood, as the concentration of Rose Bengal has been shown to rapidly decreases within the first 20 min after injection but stabilize at 40% of its original state between the 20-min to 1-h mark.^17^

## 5 Conclusion

Using the skull optical clearing window, we established a targeted, controllable ischemic stroke model without worrying about unnecessary damage or environmental changes to the brain caused by surgery to the skull. By adjusting the irradiation position and dose, the infarction region and degree can be easily controlled. Such a model holds great potential for research on ischemic stroke.
