# Supplementary material for: In vivo tissue optical clearing assisted through-skull targeted photothrombotic ischemic stroke model in mice
Source: J Biomed Opt. 2022 Jun 8;27(6):065001. doi: 10.1117/1.JBO.27.6.065001 (PMC9174889; doi:10.1117/1.JBO.27.6.065001)
Supplement: Supplementary file 1 [file JBO_027_065001_SD001.pdf]

In vivo tissue optical clearing assisted through-skull targeted photothrombotic ischemic stroke model on mice

Zhengwu Hu[a,b], Dongyu Li[a,b*], Xiang Zhong[a,b], Yusha Li[a,b], Ang Xuan[a,b], Tingting Yu[a,b], Jingtan zhu[a,b], and Dan Zhu[a,b]

[a] Britton Chance Center for Biomedical Photonics, Wuhan National Laboratory for Optoelectronics, Huazhong University of Science and Technology, Wuhan, Hubei 430074, China

[b] MoE Key Laboratory for Biomedical Photonics, Huazhong University of Science and Technology, Wuhan, Hubei 430074, China

[c] Optics Valley Laboratory, Hubei 430074, China.

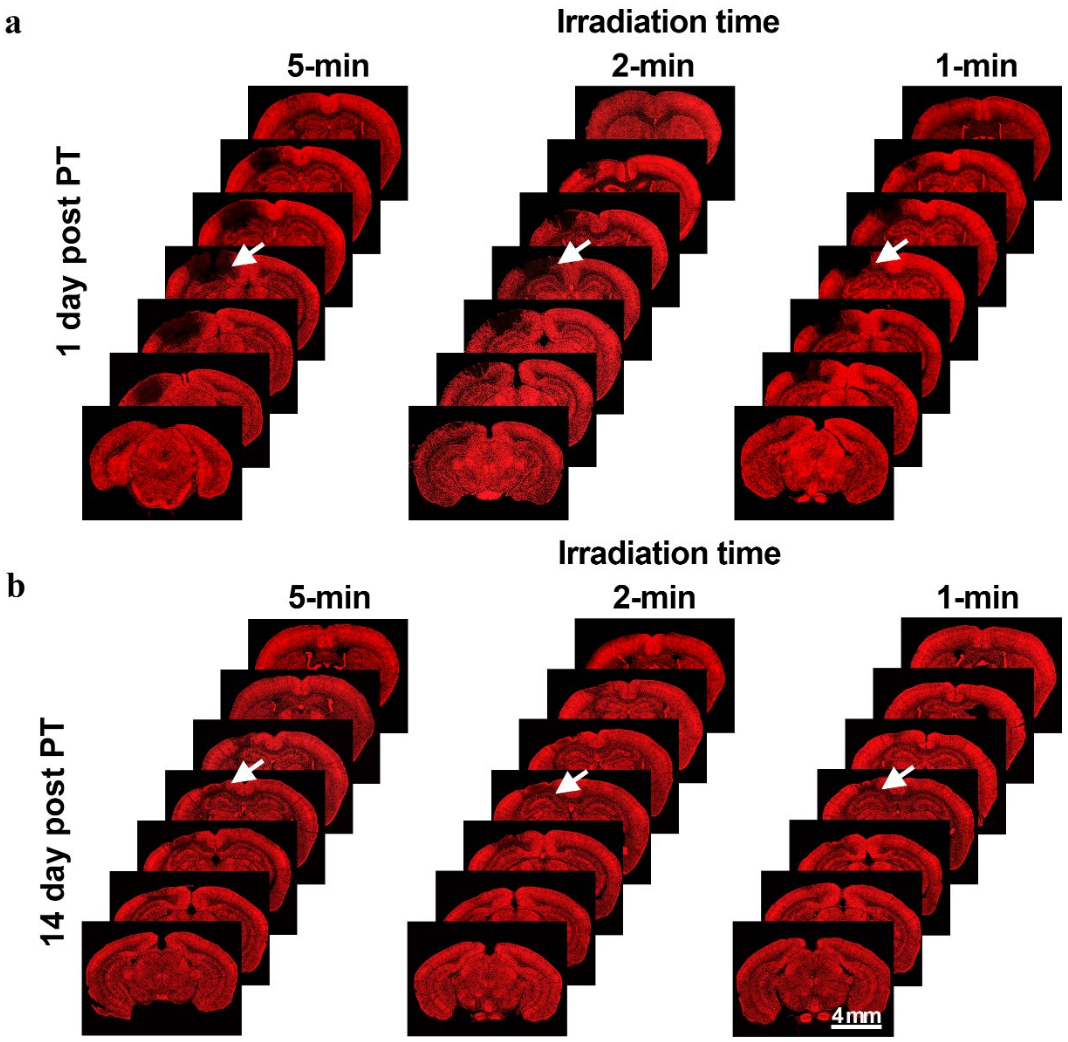

Fig S1. Representative confocal images of brain slices showing brain blood vessels (a) 1 day and (b) 14 day after photothrombosis establishment with various light doses. All spaced 500 μm apart, and the blood vessels were labeled with LEL-649 antibody. The white arrows indicate the stroke

regions.

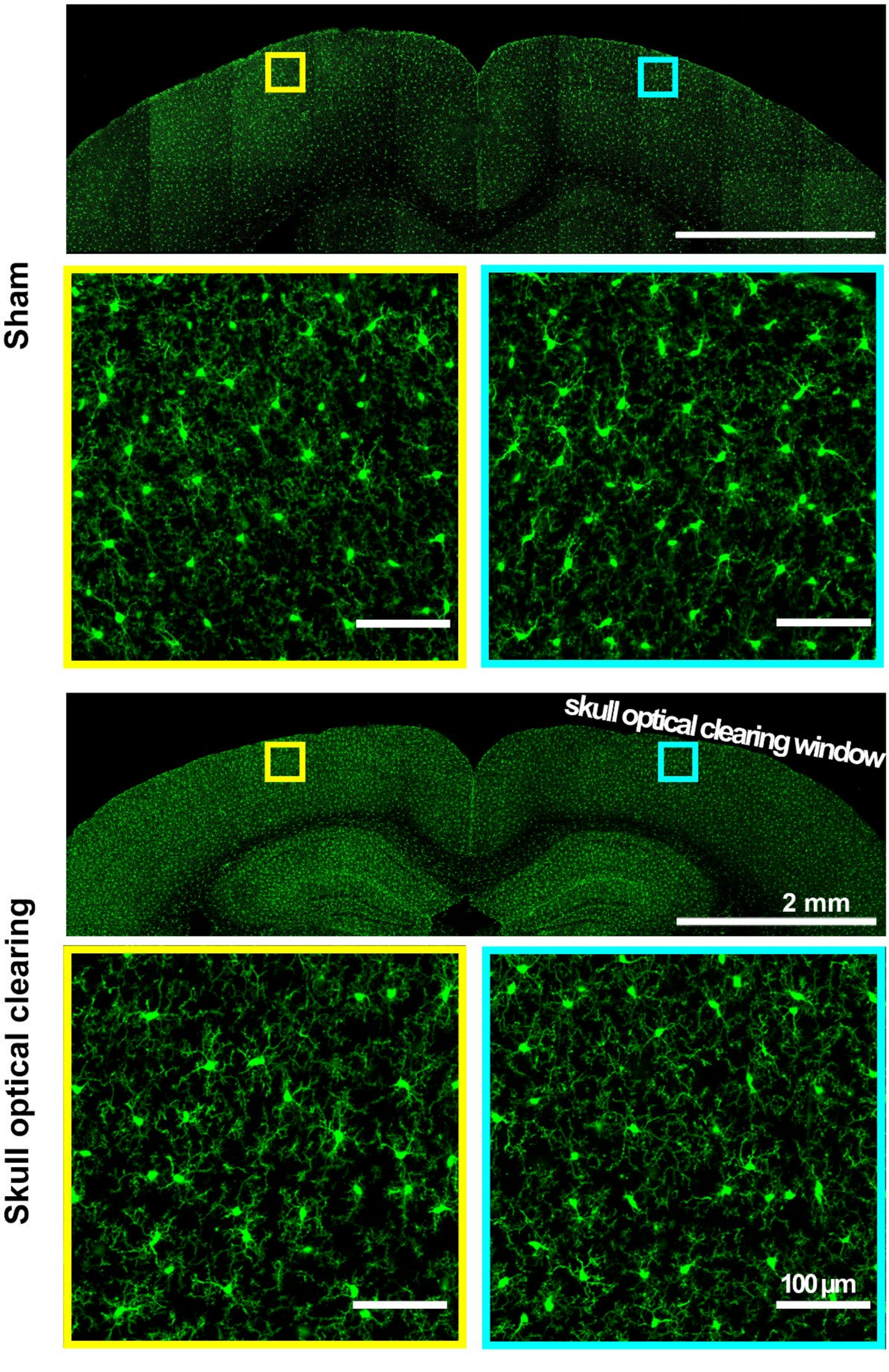
Sham
Skull optical clearing
skull optical clearing window
2 mm
100 µm

Fig. S2. Representative morphology of the cortical microglia of the mice with repeated skull optical clearing treatment. Skull optical clearing was performed on day 0, day 1, day 4, day 7 and day 14, after which they were sacrificed, and the brain was removed for sectioning. The sham group was only anesthetized without scalp-opening operation. The results indicated that the cell density, cell body size and synaptic length of microglia in the experimental group were similar to the sham group.

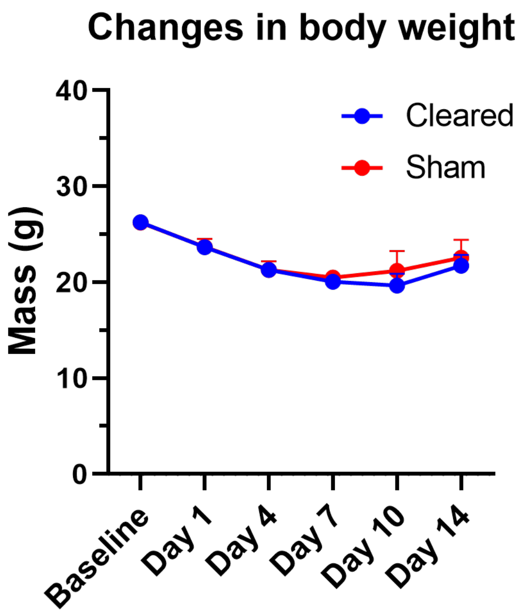

Fig S3. Weight changes in the skull optical clearing group and sham group. Skull optical clearing treatment was performed at day 0, day 1, 4, 7 and 14. The sham group was only anesthetized without scalp-opening operation. The results indicated that, there was no significant difference of body weight between the two groups, indicating the repetitive skull exposing did not lead to weight loss. Mean ± SD; n=5 per group.
